# Facilitators and barriers to using telemedicine for gender-affirming care in gender-diverse youth: A qualitative study

**DOI:** 10.1177/1357633X241231015

**Published:** 2024-02-23

**Authors:** David J Inwards-Breland, Debra Yeh, Maja Marinkovic, TR Richardson, Bixby Marino-Kibbee, Ava Bayley, Kyung E Rhee

**Affiliations:** 1 UC San Diego School of Medicine, Department of Pediatrics, La Jolla, CA, USA; 2 14444 Rady Children's Hospital, San Diego, CA, USA; 3 UC San Diego, La Jolla, CA, USA

**Keywords:** Telemedicine, transgender, gender diverse, gender-affirming care, adolescents

## Abstract

**Introduction:**

Access to gender-affirming care (GAC) is limited for gender-diverse (GD) youth, with the potential for further limitations given the current political climate. GAC has been shown to improve the mental health of GD youth and telemedicine (TM) could increase access to GAC. With limited data on the acceptability and feasibility of TM for GAC among GD youth, we sought to further explore their perspectives on the use of TM in their care.

**Methods:**

We used a semi-structured interview guide, with prompts developed to explore participants’ knowledge of TM, identify factors that influenced use, and advantages or disadvantages of use.

**Results:**

Thirty GD participants aged 13–21 years old participated in TM. While TM was not the preferred option for medical visits, it was recognized as a practical option for providing GAC. Various actual and perceived disadvantages noted by youth included, technical issues interrupting the visit, not receiving care equivalent to that of an in-person visit, having to see themselves on the screen, family members interrupting visits, and meeting new staff while connecting to a TM visit. The advantages, however, were an increased autonomy and convenience of TM, especially when used for specific aspects of GAC.

**Discussion:**

The use of TM in GAC could be optimized by limiting camera use, eliminating/reducing staff involvement, being sensitive to privacy issues, and alternating TM with in-person visits. Clinicians should be cognizant of patient preferences and concerns and be flexible with visit types.

## Introduction

Transgender stigma and discrimination often limit critical resources such as access to healthcare and affect this vulnerable population's physical and mental health, particularly gender-diverse (GD) youth.^1–3^ The proposed mechanism of how stigma can affect access to gender-affirming care (GAC), is through minority stress: distal (discrimination), and proximal minority stress (internalized transphobia) which can culminate in poor mental and physical health. Minority stress research has shown that when occurring within a healthcare setting, access to care is limited.^2–5^ Without appropriate gender-affirming mental health and medical care, many health disparities can arise. GD youth often experience significant health problems such as depression, anxiety, suicidality, substance abuse, and continued discrimination related to inadequate access to appropriate care.^6^ Moreover, the exponential rise in antitransgender youth legislation around the country has and will continue to limit access to GAC, while worsening mental health issues in this vulnerable population.^7,8^ Despite a previous increase in gender-affirming multidisciplinary programs for youth across the United States,^9^ access to care continues to be limited for GD youth, particularly in the wake of legislative bans on care.^6,7^

Medical GAC, which includes the use of gender-affirming hormone therapy and puberty blockers, has been shown to significantly improve mental health in GD youth. Tordoff et al. found a significant decrease in depression symptoms and self-harm/suicidal behavior with receipt of GAC.^10^ Turban et al. and others reported similar associations between the receipt of pubertal blockers and decreased risk of suicidal ideation.^11,12^ Given the lifesaving potential of GAC for gender dysphoria, better access to GAC is needed.

One avenue that has not been widely adopted is the use of telemedicine (TM) for GAC. Since the COVID-19 pandemic, the use of TM for many conditions including reproductive health, HIV, eating disorders, mental health, and GAC has increased.^13–21^ One study found TM to be a feasible modality to increase access for marginalized communities to vital healthcare.^20^ Youth perspectives on the use of TM have shown limited acceptability; the preferred method for health care was often in-person.^22^ However, during the peak of the COVID-19 pandemic, TM became more acceptable to youth and caregivers, and GD youth were willing to use it in the future.^15,23^ This study also determined GD youth were more willing to use TM for ongoing GAC rather than new visits.^23^

TM has the potential to decrease health disparities for GD youth by providing wider access to GAC. With limited data on the acceptability and feasibility of TM for GAC among GD youth, we sought to explore GD youth's perspectives on the use of TM in their care. We were particularly interested in their knowledge of TM, factors that influenced use (provider, staff, and parent/guardian), and the advantages or disadvantages that would influence regular TM use.

## Methods

### Study design

We conducted a qualitative study using semi-structured interviews with GD youth and young adults to explore their perspectives on TM. Individual interviews were chosen to preserve the privacy and safety of our participants. Due to the COVID-19 pandemic, these interviews were conducted via Zoom.

### Setting and participants

From April to December 2021, participants were recruited from the Center for Gender Affirming Care (CGAC) at a large children's medical center which is the primary pediatric health center for approximately 900,000 children.^24^ Our CGAC, established in 2012, has served >1200 patients to date. At the time of this study, all TM visits were provided by either medical providers or licensed clinical social workers (LCSW). Eligible youth received informational flyers in the clinic during a clinical visit and those meeting inclusion criteria were invited to participate. Inclusion criteria were: (1) self-identifying as transgender and/or nonbinary; (2) age 13–26 years; (3) receiving medical care at our center; and (4) English or Spanish speaking. Eligible participants ≥18 years completed an informed consent process in person or via Zoom. Parents/Guardians of the eligible participants <18 years completed an informed consent process, and the minor completed an assent process in person or via Zoom. Signed consents/assents were shared with the researchers via secure email. Purposive sampling was utilized (by inviting non-White/masculine identified participants) to ensure perspectives represented a wide range of ages, races, ethnicities, gender identities, and experiences within our medical center until we reached our goal of 30 participants. Participants received $25 compensation after completing the interview and survey. The study was approved by the Human Research Protections and the University of California, San Diego Institutional Review Board.

### Data collection

We used a semi-structured interview guide with prompts developed to explore participants’ experiences and perspectives on using TM for GAC (Table 1). Participants were asked open-ended questions about their knowledge of and preference for TM, perceived advantages and disadvantages, technical issues (either experienced or theoretical), and to brainstorm ways to improve the experience. All interviews were conducted by two members of the team (D.Y. and T.L.R.) via Zoom and in English (per participant preference). Interviews were concluded once the team reached thematic saturation, where no new major themes were identified. Each participant also completed a short demographic questionnaire in English. All interviews were audio-recorded, and then transcribed using a professional transcription service.

**Table 1.** Semi-structured interview questions about telemedicine.

*Telemedicine: The practice of medicine using technology to deliver care at a distance. For example, a medical provider in one location uses telecommunications (like Zoom) to deliver care to a patient at a distant site, like your home.*
1. What do you know about telemedicine? (If they do not know about it, give them the definition above) 2. Have you ever used TM for your medical or mental health care and in what way did you use it (PCP (primary care providers), subspecialist, therapist, etc.)? a. If you have never used telemedicine What do you think are the benefits of using it for your gender care? What are the reasons why you would NOT use TM for your gender care? What are reasons why you personally would use TM for your care? Can you think of situations where TM can be used for gender care? (If not already brought up) Are there any special reasons or situations where you would worry about your confidentiality? Can you tell me about any technical issues that can occur with TM? How do you feel about seeing your image on the screen if you had a TM visit? 1. If they do not like seeing their image, ask why and what could make it better. How can parent(s) help you use TM or prevent you from using TM? What are ways to increase the use of TM for gender care? What can the clinic staff do to make your experience with TM better or worse? What can the provider do to make your experience with TM better or worse? b. If you have used telemedicine Was use of TM for the visit your or provider's choice? What are the reasons why you used TM for your gender care? Tell us what you think are the benefits of using it for your gender care? What are reasons why you would NOT use TM for your gender care? Can you think of situations where TM can be used for gender care? Are there any special reasons or situations where you would worry about your confidentiality? What role did parents/guardians play in your use of TM? Was the experience better or worse for your TM visit? Can you tell me about any technical issues that can have or can occur with TM? How do you feel about seeing your image on the screen if you had a TM visit? 1. If they do not like seeing their image, ask why and what could make it better. What are ways to increase the use of TM for gender care? What did the clinic staff do to make your TM experience better or worse? What can the provider do to make your experience with TM better or worse? With the use of TM the provider cannot perform physical exam. What are the advantages and disadvantages to NOT doing a physical exam? What medical needs or care was not answered for you with TM? Would you choose TM visit in the future again, why, or why not?

### Data analysis

Interviews were deidentified and transcribed verbatim. ATLAS.ti^©^ was used for analysis. Two primary coders (D.M.Y. and T.L.R.) independently coded all transcripts using inductive thematic analysis.^25,26^ The coders met weekly and reviewed all transcripts, developed and revised the codebook, and reached a consensus on any coding discrepancies. The larger team (D.I.B., M.M., B.M.K., A.B., and K.E.R.) reviewed coding applications, categories, and themes for validation. Given the wide age range of participants, themes were reviewed by age group (13–17 years and 18–26 years old), and no variation in responses was observed. Thus, results were reported for the group at large with any themes/comments that were specific to one age group highlighted in the text. The research team was comprised of physicians experienced in qualitative research, members of the lesbian, gay, bisexual, transgender, queer, intersex, and asexual community, and providers of medical and mental health care for GD youth. Participants were later invited to review the themes through member checks to ensure an accurate representation of their perspectives. The majority (28 out of 30) expressed interest in participating in the process, and three participants responded with questions but ultimately did not want to change the findings or thematic groupings.

## Results

In total, 30 GD youth, aged 13–21 years (average 18 ± 2.15) were recruited. The majority (60%) identified as White, and 23% identified as Latine which mirrors the racial/ethnic demographics of our patient population. Most participants (50%) identified as male/transmale, 30% as female/transfemale, 13% as nonbinary, and 10% gender fluid (Table 2). Most participants preferred in-person visits (22/30).

**Table 2.** Participant demographics.

Age of participants	*N* = 30 (%)
13–17 years	14 (47)
18–21 years	16 (53)
Race and ethnicity*	
Hispanic, Latine, or Spanish origin	7 (23)
American Indian or Alaskan native	1 (3)
Asian	3 (10)
Native Hawaiian or Other Pacific Islander	0 (0)
Black or African American	3 (10)
White or Caucasian	18 (60)
Two or more races	2 (7)
Prefer not to say	1 (10)
Other	0 (0)
Gender identity*	
Female or transfemale	9 (30)
Male or transmale	15 (50)
Nonbinary	4 (13)
Gender fluid	3 (10)
Genderqueer or gender nonconforming	0 (0)
Other	0 (0)

*Total percentage exceeds 100% due to selection of multiple options by participants.

All participants knew what TM was because of its widespread use at the onset of the COVID-19 pandemic. Early on, patients had to adapt to TM if they wanted to continue to receive medical and mental health care, as in-person visits were limited. Subsequently, some participants preferred TM due to the lack of a COVID-19 vaccine and out of concern for their own safety as well as mandated by the health institutions. This participant noted:

*… it was kind of like a mutual decision because we were … still in … intense lock down. I don't, … know if like the gender Clinic was open for patients to like, [to] come in or not.*

Participants identified many advantages and disadvantages to using TM for GAC. Despite a few disadvantages, many saw a benefit to continuing the use of TM in the future (Table 3).

**Table 3.** Gender-diverse youth perspectives on telemedicine use and feasibility.

Advantages of telemedicine	
Subtheme	Quote
Convenience/ease	“…I think it's easier for people to do telemedicine, especially when they don't have the availability to carpool or to drive. … for my example, … I can't drive right now to places just cause … I don't have my license … and to drive, I have to be with someone or someone has to take me somewhere” “…you wouldn't have to leave your home. I know that much. I know you don't have to have a ride to go get care. I know a lot of kids, especially the kids who have gotten into the gender clinic themselves without parents’ help, don't have necessarily a car or ride to get there and they can't really get there on their own.” “…it's easier to access, I think. If people don't have … good transportation…” “…I just graduated … I was up at San Francisco State University. So … I couldn't be constantly flying back home for appointments every couple months. … I have animals, so I would have to drive all the way back down rather than fly with them. So [telemedicine] definitely made that-that easier.”
Autonomy/independence (all participants ≥18 years old*)	“Telemedicine would help with kids that have less supportive parents because they can, like, be in charge of, like, talking to the doctor without, you know, relying on the parents to drive them to the doctor.” “you don't have to worry about taking someone (in-person visit) who's not supportive with you.” “Definitely, if it's in confidence, I feel like that's a lot easier because if they haven't come out to a select family member that would have an issue with it, they can have their meeting in the room and still be comfortable and kind of go from there.”
More comfortable with TM visit*	“I think physical exams … are sometimes uncomfortable. So … if you're not too fond of that, … you can just do telemedicine and not necessarily … have a physical exam.” “[telemedicine is] more mellow and toned down and … you don't walk in there knowing you're going to have to do something you dislike. It's honestly just, it's kind of more, like, comfortable. [Telemedicine] less effective, but it's more comforting” “…if someone's … afraid of the doctor, then I think that [telemedicine], might be more comfortable for them.” “It's just a waste of time. … that's the benefit of a Telehealth … you don't have to do the whole like, … check-in, do vitals, all of that.” “Because some might not feel comfortable going out because of how they identify or how they look…”
**Disadvantages of telemedicine**	
Lack of perceived knowledge of a telehealth visit despite having a good working knowledge of video conferencing	“I don't really know much about it besides I went on a zoom call and they … were telling me all these things, like, to check my own weight and … see how tall I am.” “I don't know much about it but I know it's like what we're doing right now except a doctor's appointment.” “Really it's just what you would normally do on … in a doctor's visit, but through a Zoom call.”
Unable to monitor physical changes with medication	“…it's important to have the doctor … be there to make sure … there's not something wrong with you, you know.” “…you don't feel as confident in the exam as you would if you were there in person. ” “[they] couldn't really … do any physical tests like height weight or anything.”
No privacy from parents or family members at home and different from confidentiality	“I feel … people wouldn't like telemedicine because they can't get any privacy at home because [of] their family members are super nosy. And they would prefer to be able to, … their doctor in person because they know that they'll be in a separate room from their parents, and that, … there's no way that their … family members or their parents … are going to be able to, … eavesdrop.” “…I don't think I'm- not on your guys’ end but like … I mean … like, my house is fairly small and I don't know how far my voice carries or, like, if my brother's listening to this right now from the other room.”
Technical issues	“Um, wi-fi. If the wi-fi doesn't work and then it's pretty much not doable.” “…I guess like in, um, connectivity issues can happen in your house, you know, um, … Zoom possibly being down or like you have to update Zoom … someone could be late to an appointment because of that and yeah.”
Bypass ancillary staff	“I would say for me, I just prefer going straight to the doctor's just because I … want to– … get it done….”
In-person preference	“…I liked in person more … Just because I … felt more …. All right, I can’t find a word for it but I … felt more comfortable, I should say.” “…I think in general … going to appointments physically and talking to the people directly face-to-face is just what I prefer.” “[I] like the comfort of [in person] appointment. And that it's more relaxing and convenient when I'm anxious how the appointment's gonna go.” “I think in general, I like coming in … because it's nice to, … be in a place where like, I know, … the physician and I don't have to, …, be meeting new people. And also we're on our computers all day every day at this point, so any chance to get out of the house is nice.”
**Confidentiality versus privacy using telemedicine**	
No concerns around confidentiality	“I haven't had a reason yet to feel like it's not secure if that makes any sense.” “I feel pretty comfortable with … the doctors I do have appointments with. So I don't think that's really much of an issue.” “…I personally am comfortable with [telemedicine]. I live with my mom and my brother, which I'm out too and they're both supportive. I don't live with my dad … He's not, he doesn't really know about this stuff. So the only times I would feel uncomfortable would be on the rare occurrence that I'm, … at his house, like doing medicine stuff. But I mean, for me personally, I'm not super worried about it in terms of like my family members finding out”
**Future of telemedicine for gender-diverse youth**	
Provider allowing patients to turn off camera	“Probably just allowing me to … turn off my camera.” “…not having to have the camera on, is a big one, … I guess just like not being required to have it on the whole time.”
Offer telemedicine appointments	“They always ask … would you like … to use … telemedicine, or … do you want to come in person? They always ask.” “…I think just offering it as an alternative, … if someone's uncomfortable … maybe they're not cis passing and they're … afraid of being misgendered…”

* We examined results by age group and found no major differences but if there were differences that more commonly pertained to one group, we have indicated in Table.

### Advantages of TM

There were many advantages to using TM for GAC, most centered around increased practicality, and ease. Participants appreciated the convenience of TM visits, citing transportation issues (i.e., long travel times, lack of gas money, and difficulty parking) were no longer a barrier to receiving GAC. One participant noted:

*… I think it's easier for people to do, especially when they don't have the availability to carpool or to drive. … for my example, … I can't drive right now to places just cause … I don't have my license …and to drive, I have to be with someone, or someone has to take me somewhere.*

TM visits were easier for patients and their families who had busy schedules and could not spend time traveling to the clinic. Several participants also noted they were more comfortable in their homes. For older youth attending in-state colleges, TM allowed them to continue their GAC while at school.

*… I just graduated… I was up at San Francisco State University. So … I couldn't be constantly flying back home for appointments every couple month. … I have animals, so I would have to drive all the way back down rather than fly with them. So, telemedicine definitely made that-that easier.*

TM was regarded as offering more autonomy or independence to participants because it allowed them to choose the level of parental involvement with which they were comfortable. Parents/guardians of the minors could attend the entire visit or part of the visit, depending on the preference of the youth and parent. Young adults had the flexibility to conduct visits when a parent/guardian was not at home. Young adults also reflected on how TM was an effective way to see a provider for specialized GAC when they did not have supportive parents or were not out to their parents but were still living at home. This young adult noted:

*You don’t have to worry about taking someone who's not supportive with you.*

### Disadvantages of TM

As mentioned above, most participants preferred in-person visits over TM, regularly citing the comfort of an in-person visit as a top priority. These youth preferred connecting with a known provider face-to-face and put them at ease during the visit. Some GD youth noted the in-person modality helped decrease their anxiety leading up to the visit, as they were sometimes unsure how the visit would go via Zoom. However, for in-person visits, they were put at ease prior to and during the visit and it felt more personal and predictable.

*[I] like the comfort of [in person] appointment. And that it's more relaxing and convenient when I'm anxious how the appointment's gonna go.*

An in-person visit enabled more clarity when receiving their GAC and played a key role in establishing trust with their provider.

Despite having a significant working knowledge of common videoconferencing platforms such as Zoom, some participants still had limited knowledge about conducting TM visits.

*I don't really know much about it besides I went on a zoom call and they … were telling me all these things, like, to check my own weight and … see how tall I am.*

Many also did not know what to expect from the TM visit and did not enjoy the process because it reminded them of a virtual school. The experience with clinic staff assisting them to start the TM visit was thought to be an inconvenience at best, and at worst, a detriment to their care. They did not want to interact with people they did not know and felt anxious about seeing new people at the start of the TM encounter. Many patients wanted to bypass this step and see their provider directly.

*I would say for me, I just prefer going straight to the doctor's just because I … want to– … get it done*…*.*

Nevertheless, a vocal minority found the clinic staff useful in helping to navigate the Zoom platform. They appreciated assistance with logging on and ensuring that they were in the right place.

Interestingly, very few participants were concerned about technical issues with TM and most issues mentioned were theoretical. The technical issues either experienced or thought to occur were internet connection (Wi-Fi) issues and updates in the Zoom software. This participant said:

…*I guess like in, um, connectivity issues can happen in your house, you know, um, … Zoom possibly being down or like you have to update Zoom … someone could be late to an appointment because of that and yeah.*

Interestingly, many participants reported the inability to perform a physical exam was both a major advantage and disadvantage of TM. Despite the ability to defer exams in in-person visits for participants uncomfortable with their bodies, the lack of physical exams provided an opportunity to have a more comfortable visit without anxiety. This minor participant noted:

*I think physical exams … are sometimes uncomfortable. So … if you're not too fond of that, … you can just do telemedicine and not necessarily … have a physical exam.*

Nonetheless, even those who preferred no physical exam recognized the limitations of not having one. Many participants were concerned about the possibility of something going wrong with their GAC and the provider not being able to physically monitor the effects of the medication or discover the issue via TM. Moreover, GD youth did not feel confident in their own self-examinations to advise the provider of changes they were seeing. This participant noted:

*…you don't feel as confident in the exam as you would if you were there in person.*

Other disadvantages noted included the inability to assess vital signs or anthropometric measurements, conduct in-clinic blood or urine sexually transmitted disease screening, and receive vaccines.

### Confidentiality^27^ versus Privacy with using TM

A participant's experience with TM was often affected by those involved in their appointments. Outside influences came from their provider, parents, and support staff in the clinic. If they were comfortable with their provider, most participants reported no concern regarding confidentiality^a^ when using TM. They trusted the provider with the information discussed during visits and did not have any reason to feel that the TM platform was “not secure.” However, some were worried that other people/family in the house might invade their personal space during the visit.

A vast majority of the youth lived with parents/guardians at the time of the interview, yet few felt they had no privacy^b,^^28^ with their provider noted by this participant:

*… I don't think I'm- not on your guys’ end but like … I mean … like, my house is fairly small, and I don't know how far my voice carries or, like, if my brother's listening to this right now from the other room.*

Despite the concerns with privacy, there were no concerns with confidentiality as stated by this participant.

*I feel pretty comfortable with … the doctors I do have appointments with. So, I don't think that's …. much of an issue*.

### Future of TM for GAC

Overall, the GD youth felt that TM use would increase if TM appointments were offered side by side with in-person appointments. Many saw themselves alternating between TM and in-person visits, but only if they were made aware of that option when scheduling follow-up visits.

… I think just offering it as an alternative, … if someone's uncomfortable … maybe they're not cis passing and they're … afraid of being misgendered….

A vast majority of participants viewed TM as optimal for discussion-based visits with the medical provider or LCSW, such as reviewing labs, adjusting doses, and reviewing options and risks/benefits of gender-affirming surgeries. Visits with our LCSW clinicians were also viewed as more easily accessible via TM. Some participants disclosed that TM visits created feelings of anxiety and gender dysphoria because they had to see themselves on the screen.

*… not having to have the camera on, is a big one, … I guess just like not being required to have it on the whole time*.

To alleviate these concerns, they recommended patients be allowed to turn off their cameras during the appointment.

## Discussion

In recent years, TM has become a viable alternative to traditional, clinic-based care, especially in pediatric transgender medicine.^29^ For patients/families dealing with obstacles like transportation limitations, family, school, or work commitments, as well as the growing prevalence of antitransgender healthcare bans nationwide, TM offers a compelling alternative to in-person visits. Patients may need to find providers who participate in interstate licensing compacts and who may be able to provide care. In our research exploring the TM-related experiences of GD youth, the most cited advantages of TM were the convenience of visits for those who live far away from the clinic, who lack transportation, and who do not wish to have a physical examination. Our results are in line with those of Russell et al. who surveyed GD youth and caregivers during the COVID-19 pandemic and found no significant differences in communication quality, privacy, or overall satisfaction between TM and in-person visits.^30^ A similar study conducted by Kahn et al. on the advantages and disadvantages of TM and preferred visit modalities found participants liked the convenience, efficiency, and comfort in their own environment, and found TM user-friendly.^31^ In addition, these participants found TM less scary because of social anxiety consistent with our study results.^31^ Like previously published opinions of cisgender youth^15^ and transgender and nonbinary patients in Canada,^32^ most of our participants preferred in-person visits to TM. They shared that an affirming and comfortable clinic setting was favored over TM appointments.

A few participants had apprehension about their ability to maintain privacy during TM visits conducted at home and feared that their conversation with the provider may be overheard by family members. Most of our study participants, however, did not have concerns about confidentiality during TM visits. Young adults may have less concern about confidentiality since they do not need a parent/guardian to consent to treatment. Overall, most of our study subjects were supported by their parents/guardians, making confidentiality less of a concern for them. Nevertheless, GD youth and young adults without family support may have different experiences with TM use.

It is important to recognize that TM can both facilitate and hinder patient care. This was also noted by our study participants. The inability to have physical exams during the visit was cited as both an advantage and disadvantage of TM. Some participants felt reassured about the progress made toward their embodiment goals and health when examined by their doctor, while some found exams to be distressing and preferred avoiding them. Our participants favored TM appointments for discussing laboratory results and ongoing mental health therapy but wanted to alternate with in-person care. These findings deserve further exploration in larger samples of GD youth.

One of the major disadvantages of TM reported by some of our study participants was the use of the camera and viewing their image could worsen gender dysphoria. This may be specific to the GD patient population and clinicians providing GAC should be cognizant and sensitive to the patient's needs around exacerbating dysphoria. One suggestion to combat this discomfort is to reduce/eliminate “camera-on” time if requested by the patient or if the provider notes discomfort with the visit and/or suggests the patient hide their video from their screen allowing the provider the ability to see the patient. We suggest patients use emojis and avatars to help the provider understand how the patient is feeling or receiving the information they are providing and with appropriate legal, institutional policy, or insurance billing requirements.

Despite the novel information generated during these interviews, the study findings are limited by the small number of participants; however, we were able to obtain rich narratives and nuances through in-depth interviews. Selection bias may have also affected our results as we primarily recruited subjects who were already in care and who were supported by their parents/guardians. Experiences of GD youth who lack family support and access to GAC may be different leading to different views about TM. Another limitation is that most participants were White. Although this reflects the patient population seen at our CGAC, it does not reflect the diversity of the community. To lessen this limitation, we sought to obtain more diverse opinions by purposefully recruiting subjects from racial/ethnic minorities and feminine embodiment to augment those views in this study.

TM, though not a preferred choice for medical visits in our sample, has emerged as a practical option for providing GAC. The various advantages noted by youth were primarily centered around the convenience and comfort of TM when used for specific aspects of GAC. The often-cited primary areas of concern were related to being anxious about visits, encountering technical issues, and not receiving care equivalent to that of an in-person visit.

The use of TM in GAC can be optimized by limiting camera use or suggesting the patient hide their video feed on their screen, eliminating/reducing staff involvement (unless there are technical issues around logging on), being sensitive to privacy issues, and by alternating TM with in-person visits. Clinicians should be aware of patient preferences and potential concerns and work with their health systems to provide flexibility with visit types. Additional research, with a mixed-method approach and larger sample sizes, is needed to further study the utility of TM for GAC in youth and as an option for GD youth to receive care in affirming states.

## Notes

a. Confidentiality—refers to a duty of an individual to refrain from sharing confidential information with others, except with the express consent of the other party.

b. Privacy—the state of being free from unwanted or undue intrusion or disturbance in one's private life or affairs; freedom to be left alone.
